# Multi-analyte profiling of ten cytokines in South African HIV-infected patients with Immune Reconstitution Inflammatory Syndrome (IRIS)

**DOI:** 10.1186/1742-6405-7-36

**Published:** 2010-10-07

**Authors:** Catherine M Worsley, Melinda S Suchard, Wendy S Stevens, Annelies Van Rie, David M Murdoch

**Affiliations:** 1 Department of Molecular Medicine and Haematology, Faculty of Health Sciences, University of the Witwatersrand and National Health Laboratory Services, Johannesburg, South Africa; 2 Department of Epidemiology, University of North Carolina at Chapel Hill, Chapel Hill, NC, USA; 3 Department of Medicine, Duke University Medical Centre, Durham, North Carolina, USA

## Abstract

**Background:**

Immune reconstitution inflammatory syndrome (IRIS) is an important complication of HAART in sub-Saharan Africa, where opportunistic infections (OIs) including mycobacteria and cryptococcus are common. The immune system's role in HIV infected patients is complex with cytokine expression strongly influencing HIV infection and replication.

**Methods:**

We determined the expression patterns of 10 cytokines by Luminex multi-analyte profiling in 17 IRIS nested case-control pairs participating in a prospective South African cohort initiating anti-retroviral therapy.

**Results:**

Interferon-gamma (IFN-γ) expression was significantly elevated in IRIS cases compared to controls (median 9.88 pg/ml versus 2.68 pg/ml, respectively, P = 0.0057), while other cytokines displayed non-significant differences in expression. Significant correlation was observed between IL-6, IL-10, and IFN-γ expression in the IRIS patients.

**Conclusions:**

Significantly increased expression levels of IFN-γ suggest that this cytokine possibly plays a role in IRIS pathology and is a potential diagnostic marker.

## Background

HIV infection leads to a progressive loss of CD4^+ ^T cells and eventually to the onset of AIDS [1]. Highly active antiretroviral therapy (HAART) results in a dramatic reduction in AIDS-defining illnesses and mortality by inhibiting HIV replication with the subsequent recovery of CD4^+ ^T cell numbers and the restoration of immune function [2-4]. Some patients experience immune reconstitution inflammatory syndrome (IRIS), or immune restoration disease (IRD), as a result of pathological responses induced during immune restoration following the initiation of HAART [5,6]. IRIS is characterized by a paradoxical worsening of a pre-existing, or unmasking of a previously sub-clinical infection in the first weeks of HAART [7]. The immune response that causes IRIS is both excessive and unregulated, as the rapid restoration of immune function after initiating HAART leads to upregulated cell-mediated responses to live or dead infectious organisms or to antigens [2,8]. The resultant inflammation causes symptoms which can be severe. The presence of antigenic stimulus, whether infectious or non-infectious, is reportedly a pre-requisite to developing IRIS, and the incidence of IRIS is likely to be dependent on the underlying infectious burden [9,10]. In South Africa, where an estimated 5.2 million people live with HIV [11], the incidence of IRIS is reported to be 25.1 IRIS cases/100 person years [10].

The spectrum of IRIS symptoms is diverse and depends on the pathogen involved, complicating the diagnosis of IRIS [2,12]. Infectious pathogens that are often implicated in the syndrome include cryptococcus, *Mycobacterium Tuberculosis*, varicella zoster, herpes virus, Kaposi's sarcoma and cytomegalovirus (CMV) [2,9]. IRIS-associated morbidity can be considerable and may result in increased hospitalization rates, further increasing the burden of HIV in resource-poor settings where health-care facilities are already stretched to maximum capacity, particularly in countries with a high tuberculosis burden [5]. Because of the associated morbidity, and sometimes mortality, it is important to diagnose and treat IRIS in a timely fashion.

The pathogenesis of IRIS is not well understood. Although what is clinically noted is the excessive and unregulated immune response, there is no common immunological pathway and different processes seem to drive the different expressions of IRIS [2]. HIV infection itself can also provoke IRIS [5,13]. Numerous members of the cytokine network are integrally involved in regulating the replication of HIV as well as several steps of the HIV life cycle [14]. It therefore stands to reason that several cytokines may be involved in the pathogenesis of IRIS. As various cytokines possess the ability to regulate the production of other cytokines, their combined effect is often greater than the function of a single component [15]. This study was conducted to increase our understanding of the immunopathogenesis of IRIS by comparing cytokine profiles in IRIS patients and controls, and by identifying which cytokine markers contribute to the increased immune activation observed in these patients, The Bio-Plex system, which makes use of Luminex multi-analyte profiling technology allows for the analysis of many different cytokines in a single microtiter well. Using this technology, we were able to identify the cytokine profiles involved in some IRIS-related illnesses in South African patients, as well as identifying the commonalities and differences in their cytokine expression profiles.

## Methods and materials

### Study Population

This nested case-control study was a sub-study of a prospective longitudinal South African cohort monitored to determine IRIS incidence during the first 6 months of HAART treatment. HAART initiation was in accordance with the 2007 South African National Antiretroviral Treatment Guidelines, which define treatment initiation criteria as CD4^+ ^cell count ≤200 cells/ml or WHO stage IV AIDS-defining illness [10,16]. Adult patients (> 18 years) recruited in the study were HAART-naïve at the time that therapy was started at Johannesburg Hospital HIV clinics in 2006 and 2007. Enrolment into the study required willingness to provide written consent for additional blood draw and sample storage. Cases required signs and symptoms of IRIS (see IRIS case definition below). Cases and controls were HAART-duration matched in a 1:1 ratio. Ethics approval was obtained from all participating institutions. 17 case-control pairs took part in this study.

### Ethics approval

University of the Witwatersrand HREC M050347; University of North Carolina at Chapel Hill Biomedical IRB 05-1603; Duke University IRB Pro00003782.

### Immune reconstitution inflammatory syndrome case definition

IRIS is generally defined as a paradoxical clinical worsening due to a subclinical opportunistic or previously-treated pathogen as a result of an adequate response to HAART [10,17]. For 'unmasking' IRIS, a new localized infection was required from a focal inflammatory process (suppurative lymph node, pulmonary infiltrate, positive CSF culture, etc.) in a patient with no pre-existing evidence of this infection prior to HAART despite a thorough clinical and diagnostic evaluation. For the 'paradoxical' form of IRIS, a patient needed to be diagnosed and treated for an OI prior to HAART initiation. Following HAART, the patient experienced a clinical worsening (worsening lymphadenopathy or suppuration, expansion of Kaposi's lesions, recurrence of meningeal signs and symptoms) at the original or new site of infection accompanied by systemic symptoms of inflammation. In all cases, a thorough diagnostic evaluation confirmed the absence of other identifiable pathogens. For this immunological analysis, only confirmed IRIS cases (i.e. an identifiable pathogen in the setting of a documented adequate HAART response, defined as >1 log_10 _reduction in the baseline HIV RNA level) and their controls (matched for duration of HAART within a two week window) were eligible, resulting in a total sample size of 17 case-control pairs.

### Data collection and measurement of plasma cytokines

Plasma samples were collected from 17 HIV-infected IRIS cases and 17 matched controls prior to the administration of any anti-inflammatory agents. EDTA-separated plasma was stored at -20°C until analysis. Ten cytokines (IL-1β, IL-2, IL-4, IL-5, IL-6, IL-10, IL-12p70, IL-13, IFN-γ, and TNF-α) were analysed using a Human Cytokine 10-Plex Th1/Th2 assay (Bio-Rad, California, USA) and Luminex multi-analyte profiling technology (Bio-Rad, USA) according to manufacturer instructions. Plasma samples were thawed in a 37°C waterbath and diluted 1 in 4 with sample diluent, while standards were reconstituted in standard diluent. Eight standards were made in duplicate by serial dilution, with each standard being a 4-fold dilution of the previous standard. Standards and samples were incubated with the coupled magnetic beads in a multi-well plate for 1 hour at room temperature. Following this, detection antibody was added to each standard and sample, and a further 30 minute incubation period was observed. Streptavidin-PE was used as the fluorochrome for antibody detection. Using Bio-Plex Manager software version 5.0, standard Luminex maintenance procedures were performed, and Bio-Plex CAL1 and CAL2 beads were used to calibrate the system. A new protocol was prepared and standard information was entered for each cytokine tested. Sample information was entered; all standards and samples were assayed concurrently, on the same plates, in order to avoid intra-assay variability.

### Statistical analysis

Median cytokine expression levels of IRIS cases were compared to non-IRIS controls. Nonparametric Spearman correlation was applied to quantify the relationships between IFN-γ, IL-6, and IL-10 cytokine responses. P values less than 0.05 were considered significant. All statistical analyses were performed using GraphPad Prism version 4.0 for Windows (GraphPad Software, San Diego, California, USA).

## Results

### Description of study participants

As reported previously by Murdoch *et al. *(2009), IRIS cases had a significantly lower baseline CD4^+ ^count at the initiation of HAART compared to matched non-IRIS controls (79 versus 132 cells/mm^3^, respectively, P = 0.02). This is in keeping with other studies where low baseline CD4^+ ^T-cell counts were a risk factor for developing IRIS [10,18]. HIV RNA levels at baseline and at sampling were similar between IRIS and non-IRIS control groups [17]. This is unexpected as viral load usually correlates with immune activation which potentially feeds cytokine production [19]. The median time interval between HAART initiation and the development of IRIS was 38 days (interquartile range 24-56 days), with blood sampling for immunological analysis occurring on average one week after IRIS diagnosis and clinical evaluations were complete.

IRIS cases exhibited a range of manifestations. These included nontuberculous lymphadenitis (n = 1), follicular facial rash (n = 1), bacterial scalp abscess (n = 1), genital herpes (n = 1), lip zoster (n = 2), abdominal TB (n = 2), Kaposi's sarcoma (n = 1), pulmonary TB (n = 3), TB adenitis (n = 2), and cryptococcal meningitis (n = 3).

### Plasma pro- and anti-inflammatory cytokine concentrations differ between IRIS patients and non-IRIS controls

**Figure 1:**
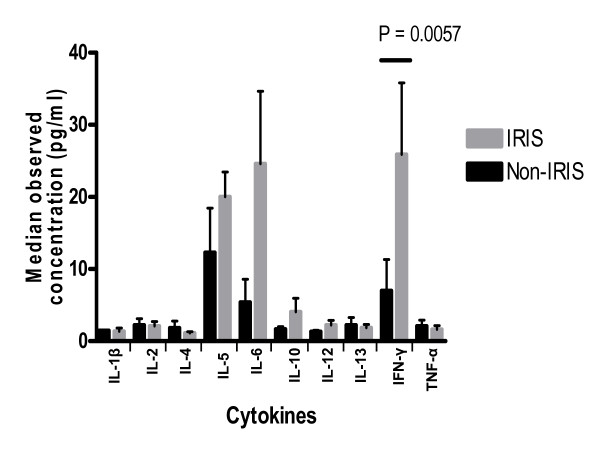
**Levels of relative changes in cytokine expression in IRIS patients compared to HAART-duration matched controls**. Nonsignificant elevations in expression levels are seen in IL-5, IL-6, and IL-10, whereas IFN-γ expression is significantly increased in IRIS patients. The other cytokine expression levels do not vary noticeably between IRIS patients and controls.

Luminex analysis revealed differences in the cytokine concentration levels between IRIS cases and non-IRIS controls within each disease manifestation. IL-1β was below the levels of detection of the Luminex and was excluded from further analyses. Overall, most IRIS cases exhibited significantly increased IFN-γ expression compared to non-IRIS controls (median 9.88 pg/ml versus 2.68 pg/ml, respectively, P = 0.0057) (Figure 1). Most IRIS cases also demonstrated increased but non-significant elevations of IL-6 (IRIS case median 11.38 pg/ml versus Non-IRIS 2.80 pg/ml, P = 0.2114) and IL-10 (IRIS case median 1.21 pg/ml versus Non-IRIS 1.02 pg/ml, P = 0.5751) (Figure 1). Marginal and non-significant increases in expression were detected in IL-5 and IL-12, while slight decreases were noted in IL-1β, IL-2, IL-4, IL-13, and TNF-α in IRIS patients (Figure 1). In general, most IRIS patients showed an increase in IL-6 and IFN-γ in comparison to non-IRIS controls (see Table 1).

**Table 1:** Relative cytokine expression patterns of opportunistic infection-related IRIS cases compared to matched controls.

Infectious disease	IL-1β	IL-2	IL-4	IL-5	IL-6	IL-10	IL-12	IL-13	IFN-γ	TNF-α
**Nontuberculous lymphadenitis (n = 1)**	E	D	E	E	E	E	E	D	I	E

**Follicular facial rash** **(n = 1)**	E	E	E	I	E	D	E	E	E	E

**Bacterial scalp abscess (n = 1)**	E	E	D	E	I	E	E	D	D	D

**Genital herpes (n = 1)**	E	E	E	D	E	E	E	E	D	E

**Lip zoster** **(n = 2)**	E	E	E	E	I	E	E	E	I	E

**Abdominal TB (n = 2)**	E	E	E	E	I	E	E	E	I	E

**Kaposi's sarcoma (n = 1)**	E	E	E	E	I	I	I	I	I	D

**Pulmonary TB (n = 3)**	E	E	E	I	I	E	E	E	I	I

**TB adenitis** **(n = 2)**	E	E	E	E	I	I	E	E	I	E

**Cryptococcal meningitis** **(n = 3)**	E	E	E	I	I	E	E	E	I	E

Each disease manifestation exhibited a unique cytokine expression profile. I = relative increases in cytokine expression in IRIS cases compared to Non-IRIS controls, E = equivocal expression compared to Non-IRIS controls, and D = relative decreases in expression compared to Non-IRIS controls.

When comparing cytokine levels between IRIS cases and non-IRIS controls by the type of IRIS presentation, different cytokine expression patterns were observed but numbers of cases were too small for statistical sub-group analysis. While most IRIS cases showed an increase in IL-6 and IFN-γ in comparison to non-IRIS controls (see Table 1), it is evident that each opportunistic illness exhibited a unique cytokine profile. IRIS cases with bacterial scalp abscess or genital herpes had decreased IL-6 compared to their non-IRIS controls. In the IRIS patient that presented with Kaposi's sarcoma, increases in IL-6, IL-10, IL-12, IL-13 and IFN-γ expression were shown (Table 1). All TB patients had increases in IL-6 and IFN-γ, with TB adenitis cases also showing an increase in IL-10 expression. Pulmonary TB also showed and increase in IL-5 and TNF-α. Although it seemed that both IL-6 and IFN-γ were increased in most IRIS patients in comparison to non-IRIS controls, only IFN-γ showed a significant difference in expression between IRIS patients and non-IRIS controls (Figure 1).

**Figure 2:**
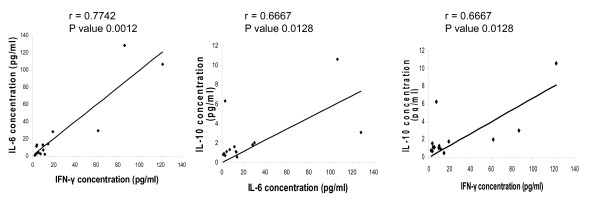
**Correlation between IL-6, IL-10, and IFN-γ**. The correlations between IL-6, IL-10 and IFN-γ concentrations in IRIS patients were significant.

Given the likelihood that complex cytokine expression profiles are present in immunological responses such as IRIS, correlation analyses were performed. Within IRIS cases, there were significant correlations between IL-6, IL-10 and IFN-γ expression over the range of conditions studied (P < 0.05) (Figure 2).

## Discussion

It is difficult to try and postulate the mechanisms that could lead to clinical IRIS, as the balance between pro- and anti-inflammatory cytokines is at the root of both HIV disease and IRIS. In this immunological analysis, IFN-γ expression was significantly elevated in IRIS cases compared to non-IRIS controls matched for HAART duration. This observation is in accordance with what has previously been reported in that the initiation of HAART results in a shift from a Th2 to a Th1 cytokine profile, which may lead to an increase in IFN-γ expression [20]. Elliot *et al. *2009 also observed greater increases in IFN-γ in TB-IRIS patients compared with non-TB-IRIS controls on HAART. IFN-γ expression was significantly increased in the IRIS patients in our study in the majority of cases, most notably in those exhibiting nontuberculous- and TB-lymphadenitis, lip zoster, abdominal or pulmonary TB, Kaposi's sarcoma, and cryptococcal meningitis. This increase in IFN-γ may be due to increased numbers of circulating T-cells that produce IFN-γ when they are stimulated with pathogen-specific antigens, which has previously been reported in patients with TB or cryptococcal IRIS [5,15].

Although this study was limited in terms of small sample number, some heterogeneity in cytokine responses was observed between the different IRIS OIs. We hypothesize that this may indicate a host-pathogen interaction in determining the immunopathology of IRIS. In addition, we observed an elevation of IL-6 expression in most IRIS cases. IL-6 is believed to play a role in the development of the inflammatory response during immune restoration and may act as a marker for persistent immune activation [21]. In our study, IRIS cases produced higher amounts of IL-6 than non-IRIS controls, but it is unclear whether this is a consequence or a cause of the IRIS.

IL-10 expression was also increased in IRIS cases compared to non-IRIS controls, although this observation did not reach statistical significance. IL-10 has many suppressor functions including the inhibition of pro-inflammatory cytokine production and the inhibition of dendritic cell expression of co-stimulatory molecules [6]. IL-10 is known to suppress IFN-γ production and immune responses to mycobacterial antigens [22] and has been postulated to be deficient in IRIS individuals, leading to a Th1 predominant cytokine profile responsible for the clinical manifestations of IRIS [18]. Impairment of IL-10 production despite an expansion of T regulatory cells (Tregs) has also been observed in immune responses to atypical mycobacterial antigens [23]. Our finding of increased IL-10 levels in IRIS patients may suggest that the pathogenesis of IRIS is not simply due to a scarcity of immunoregulatory cytokines resulting in an overactive inflammatory response. Rather, this observation could suggest that the exuberant pro-inflammatory antigenic response that characterizes IRIS occurs first, followed by a compensatory increase in IL-10.

This is an interesting observation in light of the recent reports of expanded numbers of regulatory T cells (Tregs) in HIV [24-29]. While there are some scenarios that would incorporate our understanding of immune activation as a hallmark of progressive HIV infection together with the inflammatory manifestations of IRIS [30], our finding of elevated IL-10 supports the hypothesis that a vigorous pro-inflammatory response is the primary trigger of both the symptoms of progressive disease as well as IRIS manifestations. Elevations in regulatory cells and cytokines would be a compensatory secondary event. Also, as regulatory T cells are thought to act largely via cell-cell interaction rather than cytokine secretion, interpretation of IL-10 as a suppressor cytokine may be limited to certain subsets of Tregs (such as induced, rather than natural Tregs) [30,31].

The correlation between IL-6, IL-10, and IFN-γ may be due to common pathways of action. IL-6 and IL-10 both signal through the JAK1-STAT3 pathway, although there is a lack of understanding of how specificity in gene expression is determined by the two different receptors [32,33]. IL-10 reportedly functions to inhibit the inflammatory responses from activated macrophages and dendritic cells, and may even prevent the clearance of pathogens [32,33]. Yet other STAT3-activating receptors, such as IL-6, do not seem to activate the same anti-inflammatory response [33]. This may be because the IL-10 receptor activates STAT3 in a SOCS3 (suppressor of cytokine signaling 3)-independent manner, while IL-6 receptor activation of STAT3 requires SOCS3 modulation [33]. SOCS3 seems to be one of the regulatory molecules in the process of JAK1-STAT3 activation, but better delineation of the JAK-STAT signaling pathway is needed to fully understand how different cytokines control the expression of different genes through this signaling pathway.

This immunological study had a number of limitations. Although in the initial study we recruited one of the largest prospective IRIS cohorts to date, the overall number of confirmed IRIS cases was limited with only a few case-control pairs in each subgroup analysis. To better characterize potential cytokine-specific immune responses in IRIS patients, larger sample sizes would be needed in future studies. We were further limited by the lack of longitudinal immunological sampling, and it is important to note differences in cytokine profiles may be indicative of the timing of immunological sampling rather than a true difference. Future immunological studies should collect samples over time and accurately describe these intervals to allow suitable comparisons and interpretation. This would contribute to our understanding of the syndrome, since immune reconstitution is a dynamic and not a static observation.

Lastly, while Luminex-based serum assays provide a wealth of cytokine expression data, we were unable to conclude which cell types are responsible for our observations. It is likely that a number of cell types are involved in the production of cytokines, as immune activity during HIV infection involves the activation and proliferation of most immune cell types [19]. Ideal IRIS immunological studies should also employ flow cytometric assays, such as intracellular staining and stimulation assays to better elucidate the immunopathogenesis.

## Conclusions

Consistent with other studies, we observed an increase in IFN-γ production in most individuals experiencing IRIS. The significant IFN-γ increase across most IRIS manifestations suggests its potential use as an overall adjunct diagnostic marker for the syndrome. However, whether the measurement of additional cytokines is useful within specific disease presentations remains unknown. Further investigation into additional cytokine measurements may lead to a better understanding of disease-specific manifestations of IRIS, leading to improved diagnosis and management of this complex condition.

## Abbreviations

AIDS: acquired immunodeficiency syndrome; CMV: cytomegalovirus; EDTA: ethylenediaminetetra-acetic acid; HAART: highly active anti-retroviral therapy; HIV: human immunodeficiency virus; IFN-γ: interferon-gamma; IL: interleukin; IRD: immune restoration disease; IRIS: immune reconstitution inflammatory syndrome; OI: opportunistic infection; PE: phycoerythrin; RNA: ribonucleic acid; SOCS: suppressor of cytokine signalling; TB: tuberculosis; TNF-α: tumour necrosis factor alpha; Treg: T regulatory cell.

## Competing interests

The authors declare that they have no competing interests.

## Authors' contributions

CMW performed all the laboratory work, statistical analysis, and prepared the manuscript. MSS aided in preparing the manuscript. WSS reviewed the manuscript. AVR assisted with study design and reviewed the manuscript. DMM designed the study and cohort, and aided in preparing the manuscript. All authors read and approved of the final manuscript.
